# Supplementary material for: Intensity interferometry for holography with quantum and classical light
Source: Sci Adv. 2023 Jul 5;9(27):eadh1439. doi: 10.1126/sciadv.adh1439 (PMC10321744; doi:10.1126/sciadv.adh1439)
Supplement: Supplementary file 1 — Supplementary Materials Figs. S1 to S4 Table S1 [file sciadv.adh1439_sm.pdf]

Science Advances
AAAS

Supplementary Materials for

Intensity interferometry for holography with quantum and classical light

Guillaume Thekkadath *et al.*

Corresponding author: Guillaume Thekkadath, guillaume.thekkadath@nrc.ca

Sci. Adv. **9**, eadh1439 (2023)
DOI: 10.1126/sciadv.adh1439

This PDF file includes:

Supplementary Materials
Figs. S1 to S4
Table S1

Supplemental Material

Interference visibility

In Eq. 2, we showed that the visibility of intensity interference depends on the photon-number statistics of the signal and reference, i.e. $V_0 = 2/A$ where $A = 2 + g_s^{(2)}/\epsilon + \epsilon g_r^{(2)}$. That derivation assumed the interfering fields occupied the same temporal-spectral mode, had identical polarisation, and were measured by ideal detectors. Rather than individually modeling each possible imperfection (e.g. see Ref. (*9*) for a treatment of temporal distinguishability), we can simply model their combined effect to the visibility by introducing a phenomenological parameter M:

$$V = MV_0 = \frac{2M}{2 + g_s^{(2)}/\epsilon + \epsilon g_r^{(2)}}. \tag{S1}$$

We determine M empirically using our observed interference visibilities. In Fig. S1, we plot Eq. S1 using the measured $g^{(2)}$ values (i.e. $g_r^{(2)} = 1.006(11)$ and $g_s^{(2)} = 0.049(8)$, $1.006(11)$, $1.920(6)$ for the single photon, coherent, and thermal signal, respectively). We find reasonable agreement with our data points [markers] using $M = 0.3$ and no other fitting parameters. In the next section, we describe the contribution of various imperfections to M.

Imperfections contributing to M

We present various measurement to give a rough idea of the relative contribution of each imperfection to the reduced visibility. The results are summarized in Table 1.

Imperfection	Estimated reduction to V
Mode overlap	85%
Camera noise	70%
Camera temporal resolution	95%
Camera spatial resolution	50%
Total	$M \sim 30\%$

Table S1: Estimate of the contribution of various imperfections to the reduced visibility V.

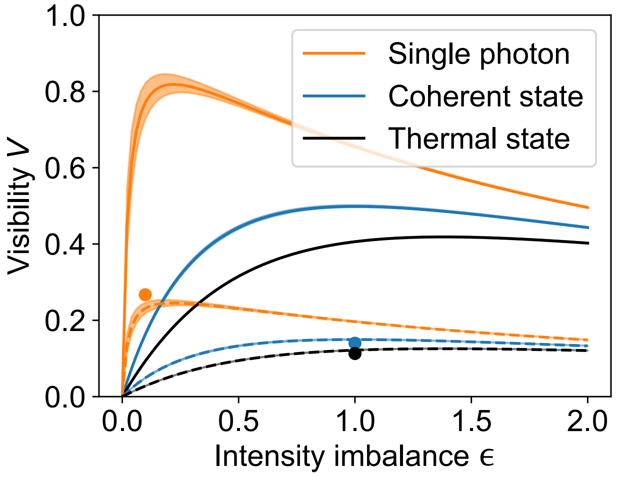

Figure S1: **Visibility**. Visibility V [Eq. S1] of intensity interference as a function of the average input intensity imbalance $\epsilon = \langle \hat{I}_r \rangle / \langle \hat{I}_s \rangle$. Continuous lines use $M = 1$ while the dashed lines use $M = 0.3$. The line thickness shows one standard deviation of uncertainty due to the measurement error in $g_r^{(2)}$ and $g_s^{(2)}$. Markers are experimental data points.

Mode overlap and camera noise

We characterized the mode overlap (i.e. polarization and temporal-spectral) between the heralded single photon signal and coherent state reference using a Hong-Ou-Mandel-type measurement. The setup is shown in Fig. S2A. Using a flip mirror (FM), we switched between measuring the interference using APDs (path 1) or the camera (path 2). The threefolds measured with the APDs [blue crosses] are plotted in Fig. S2B as a function of the delay τ_1. We observe a peak rather than the conventional dip since we are detecting bunching at the output port of PBS3. The peak has a visibility of $V = 84(2)\%$, where $V = (C_{\max} - C_{\min})/C_{\max}$ and $C_{\max}$ and $C_{\min}$ are the maximum and minimum count rate obtained from a Gaussian fit. The observed visibility is smaller than the upper limit given by the $\sim$92% spectral modal purity of the SPDC source inferred from the unheralded second-order autocorrelation measurement, $g^{(2)} = 1.920(6)$ (*23*).

We then switch to measuring this interference signal with the camera. In this case, we treat

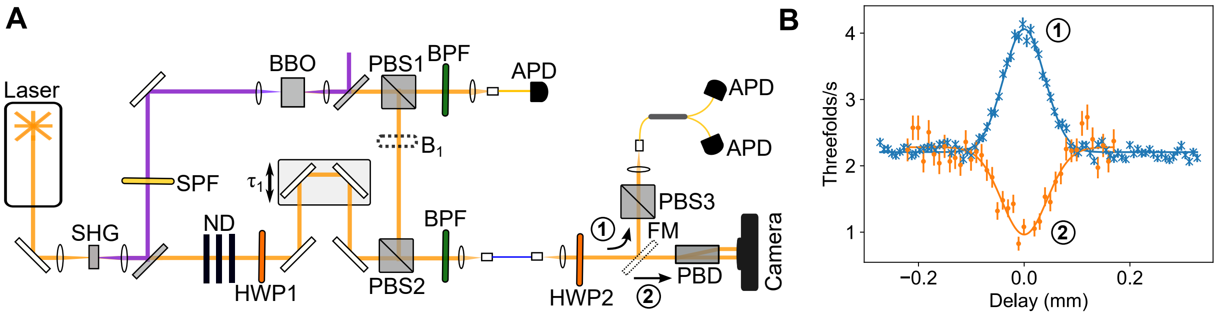

Figure S2: **Hong-Ou-Mandel-type interference**. (A) Experimental setup used to characterize the interference visibility using APDs (path 1) or the camera (path 2). The signal is a heralded single photon state, while the reference is a weak coherent state. (B) Observed peak (dip) in the measured threefolds as a function of the delay τ_1 measured by the APDs (camera). The visibility of the peak (dip) is 84(2)% (58(4)%).

the camera region at each output of the PBD as a bucket detector. The threefolds [orange dots] are plotted in Fig. S2B and show a dip with a visibility of 58(4)%. We attribute the lower interference visibility measured by the camera to three main factors: (i) increased dark counts ($\sim 5 \times 10^4$ counts/s) (ii) reduced temporal resolution (see below), (iii) increased multiphoton noise. This last effect is related to the lower detection efficiency of the camera ($\sim 7\%$) compared to the APDs ($\sim 60\%$): in order to achieve a similar threefold count rate when using the camera, we increased the SPDC pump power and reference intensity, which increases the multiphoton noise.

We note that the imperfect mode overlap should not contribute to M for the coherent state signal, since in this case, both the signal and reference fields are derived from the same source.

Camera temporal resolution

The finite temporal resolution of the camera causes accidental correlation events which also contribute to reducing V. Their origin can be seen in Fig. S3. The blue line is a histogram of the timestamp difference between a photon detected by the camera (in either the left or right beam region) and a herald photon detected by the APD. One can observe a train of pulses

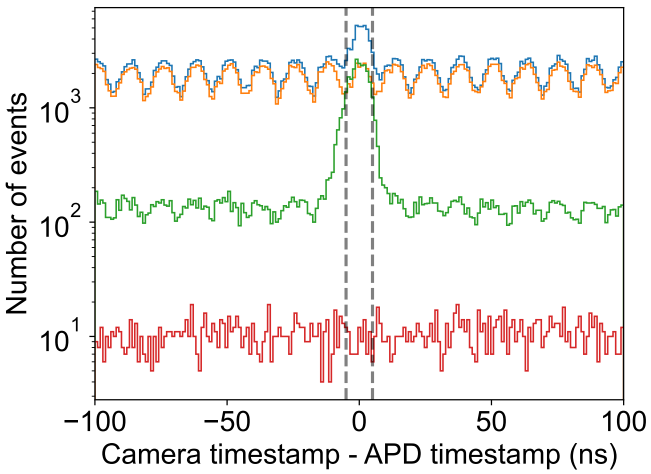

Figure S3: **Timestamp histogram**. Difference between the timestamp produced by a herald photon arriving at the APD and a photon arriving at the time-tagging camera in 10 s of integration. The signal is a heralded single photon and the reference is a coherent state. Blue: both signal and reference unblocked, orange: signal blocked, green: reference blocked, red: both blocked. Dashed grey lines show the correlation window $\tau_w = 5$ ns.

separated by 12.5 ns (corresponding to the repetition rate of the laser) with a peak near $t = 0$ due to the correlated photon pairs produced by SPDC. While the pulses themselves are only picoseconds in duration, their shape is convolved by the camera response time $\tau_d \sim 8.3(6)$ns. Thus, photons from different pulses have a chance to produce timestamps within the correlation window, which contribute to accidentals. By fitting Gaussians to the first three peaks around $t = 0$, we estimate that these accidentals correspond to roughly 2% of the measured threefolds for a correlation window of size $\tau_w = 5$ ns. In the same figure, we show histograms obtained with the heralded photon blocked (orange), the coherent state blocked (green), and both blocked (red).

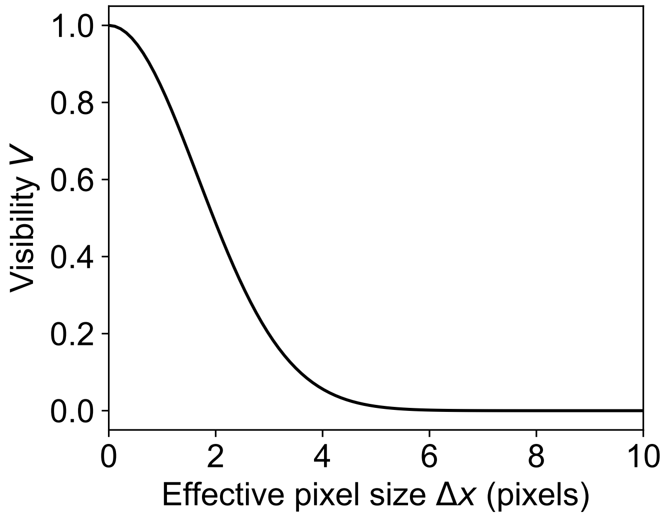

Figure S4: **Visibility and spatial resolution**. The observed visibility V of the intereference pattern diminishes as the effective pixel size of the camera Δx increases. Here we assumed the interference fringe period is given by $2\pi/k_0$, where $k_0 = 0.62$ [1/pixel] is shear strength used in the experiment.

Camera spatial resolution

There remains a substantial discrepancy between the 58(4)% visibility measured in Fig. S2(b) compared to the 26.6(4)% measured in Fig. 3. We attribute the remaining reduction in V to the finite spatial resolution of the camera. In the latter measurement, we introduced a shear between the signal and reference beams in order to use off-axis holography. The shear had a strength of $k_0 = 0.62(2)$ [1/pixels] leading to an interference pattern with a fringe period of $2\pi/k_0 \sim 10$ pixels. Due to the camera intensifier, a single photon causes a cluster of pixels to fire, typically containing 6-7 pixels (*49*). We assigned the pixel with the largest response (i.e. time-over-threshold) as the spatial coordinate for that detection event. This leads to a blurring effect which reduces the effective resolution of the camera. We model this effect by convolving the expected fringe pattern with a Gaussian of standard deviation Δx [Fig. S4]. An effective pixel size of $\Delta x \sim 2$ pixels accounts for the remaining discrepancy in the observed visibility.
